# Exploring the Effect of Augmented Reality on Cognitive Load, Attitude, Spatial Ability, and Stereochemical Perception

**DOI:** 10.1007/s10956-022-09957-0

**Published:** 2022-01-28

**Authors:** Daniel Elford, Simon J. Lancaster, Garth A. Jones

**Affiliations:** School of Chemistry, University of East Anglia, Norwich Research ParkNorwich, NR4 7TJ UK

**Keywords:** Augmented reality, Game-based learning, VSEPR, Undergraduate, Problem solving, Interactive visualisation

## Abstract

Augmented reality (AR) has the capacity to afford a virtual experience that obviates the reliance on using two-dimensional representations of 3D molecules for teaching stereochemistry to undergraduate students. Using a combination of quantitative instruments and qualitative surveys/interviews, this study explored the relationships between students’ attitudes, perceived cognitive load, spatial ability, and academic performance when engaging in an asynchronous online stereochemistry activity. Our activity was designed using elements of game-based learning, and integrated AR technologies. The control group was provided with a copy of our activity that used two-dimensional drawings, whereas the AR group completed an activity using the AR technologies. For this cohort of students, results indicated significant improvement in academic performance in both the control and AR groups. The introduction of AR technologies did not result in the AR group outperforming the control group. Participants from both groups displayed significant improvements in spatial ability throughout the research period. Further, a moderate correlation (*r*_*s*_ = 0.416) between students’ spatial ability and academic performance was found. No significant intergroup differences in the perceived cognitive loads of students were observed. A significant difference was observed on one item of the Intellectual Accessibility subscale of the ASCI (V2), Complicated–Simple. We found no correlation for student attitude or cognitive load with academic performance. The findings of this study provide insights for future AR-related studies to explore the role of spatial ability, student attitude, and cognitive load in learning performance.

**Supplementary Information:**

The online version contains supplementary material available at 10.1007/s10956-022-09957-0.

## Introduction

### VSEPR and Learning With Augmented Reality

The Valence Shell Electron Pair Repulsion (VSEPR) theory is an archetypical example of stereochemistry, a model in chemistry that provides an explanation for the basic geometry of many main group compounds encountered by chemistry students based upon the extent of electrostatic repulsion. The “AXE method” of electron counting is commonly applied to determine the shape of a molecule based on the principles of VSEPR (Burrows et al., 2021). The “A” represents the central atom. The “X” represents *m* number of bonds between the central atom and its substituents. The “E” represents *n* number of lone pairs surrounding the central atom. The sum of *X* and *E*, obtained from a molecule’s Lewis structure, are denoted as the steric number.

In AX_m_E_n_ molecules, electrostatic interactions repelling volumes of negative charge leads to the formation of a most-probable spatial arrangement. Take one example, sulfur hexafluoride, that has a most-probable octahedral shape to maximise the distance between the fluorine substituents to reach an energetic minimum (Gillespie, 1963). Visualising the three-dimensional molecular shapes requires cognitive processes in the spatial domain, and thus, it is crucial that students can mentally perceive/visualise them. Consequently, educators are increasingly introducing a variety of instructional media and resources to teach the principles of VSEPR. Previous examples include approaches embedding elements of gamification and molecular model building (Erlina et al., 2018), molecular computer modelling and the use of experimental data (Martin et al., 2015; Pfennig & Frock, 1999), and 3D printing technology (Dean et al., 2016).

An upcoming approach is the integration of augmented reality (AR) technologies into the teaching and learning process. AR is a technique that imposes computer-assisted contextual information onto the physical world (Milgram et al., 1995), obviating the reliance of using 2D representations of 3D molecules, as is the case when using hardcopy textbooks. No longer does an educator need to make arbitrary judgements about the most effective representation to carry the learning objective. This initiative liberates the two-dimensional constraints of a representation, and places control into the fingertips of the individual student, promoting active learning in the affective and cognitive domains (An & Holme, 2021; Keller et al., 2021).

### Cognitive Load

Cognitive load theory posits that people learn best under conditions that align with human cognitive architecture (Sweller et al., 1998). Learning requires working memory resources to process new information and is assumed to have a limited capacity regarding the amount of information that can be processed simultaneously. In addition, not everyone experiences cognitive load in the same way (Kalyuga, 2007). Therefore, instructional methods should avoid exceeding these limitations to avoid cognitive overload (Sweller et al., 2019).

Instruction can impose three types of cognitive load on learners: intrinsic cognitive load (ICL) determined by task complexity and learners’ prior knowledge, extraneous cognitive load (ECL) determined by instructional features that are not beneficial to learning, and germane cognitive load (GCL) determined by instructional features that are beneficial to learning (Sweller et al., 1998; van Gog et al., 2010; Van Merriënboer & Sweller, 2005). In recent years, researchers have suggested a modified dual model that includes only ICL and ECL and gives a broader interpretation to ICL, depending on the goals of learning and instruction (Leppink, 2017). It is important to note that this dual model does not deny the existence of GCL and that the two models support the same guidelines for the design of education (Leppink, 2017).

Specific recommendations regarding instructional design show that ICL should be optimised by selecting learning tasks that match learners’ prior knowledge and experience (Kalyuga, 2009), but that high ICL can lower learning outcomes (Ayres, 2006). In addition, ECL should be minimised to reduce ineffective load (Kalyuga & Hanham, 2011; Paas et al., 2003). When ICL is optimal and ECL is low, learners can engage in knowledge elaboration processes that impose GCL and facilitate learning.

### Spatial Ability and Attitudes to Study

Spatial ability is required to produce and mentally manipulate abstract images (Harle & Towns, 2010). It is related to an individual’s capacity to understand the shape and position of objects visually (Carlisle et al., 2015). It has been widely recognised that spatial ability is an important contributor to the successful learning of scientific principles and academic performance (Carlisle et al., 2015; Charlesworth et al., 2005). Bodner and Guay (1997) showed a highly significant corelation between spatial ability and spatially orientated tasks in general chemistry. Yet, spatial ability is not a skill that is taught explicitly by STEM educators and has been demonstrated to be capable of improvement over time through practice (Rahmawati et al., 2021; Yang et al., 2003). In chemistry-related disciplines, students should be able to translate representations of molecular shapes, as well as visualising them from different perspectives.

The understanding of subject content falls under the cognitive domain, whilst students’ attitudes fall under the affective domain. Thus, giving consideration to students’ attitudes and learning experiences can help ensure quality in teaching and learning. The ideal curriculum is one that supports both gains in content knowledge and positive attitudes towards the study of science.

Previous studies have highlighted the correlation between attitude and academic achievement. A low correlation has been previously reported (Brown et al., 2015), suggesting that achievement is independent of students’ attitudes. Others claim that a non-cognitive factor such as attitude is a predictor of chemistry achievement (Kahveci, 2015; Xu et al., 2013). The two-factor subjective test instrument utilised in this study (ASCI (V2)) is designed to measure a student’s “intellectual accessibility”. This is influenced by their levels of prior knowledge in chemistry (Xu et al., 2013). Results from prior research have demonstrated an interrelationship between previous chemistry academic achievement and students’ intellectual accessibility and emotional satisfaction towards chemistry (Kahveci, 2015). Such findings have an important implication for educators, as students’ achievement in chemistry may be improved by not only building conceptual knowledge, but by also reinforcing a positive attitude to the study of chemistry.

## Methods

### Test Instruments

The following test instruments were employed throughout this study:

#### Cognitive Load Scale

Students’ cognitive load was measured via an adapted version of the Cognitive Load Scale (CLS) (Leppink et al., 2013). The CLS is a previously validated three-component psychometric instrument considered capable of distinguishing between intrinsic, extraneous, and germane load (Hadie & Yusoff, 2016). This scale develops upon previous unidimensional tools that measure total cognitive load such as Paas’ (1992) nine-point scale, helping researchers to determine the efficacy of learning environments as a function of instructional format and learner characteristics. The scale was adapted to the context of the VSEPR learning activity (see Appendix 1).

#### The Attitude to the Study of Chemistry Inventory

Student’s attitude to the study of chemistry was measured using the ASCI (V2) developed by Xu and Lewis (2011). The ASCI (V2) is an 8-item refinement of the original 20-item semantic differential scale developed by Bauer (2008). It measures two factors: Emotional Satisfaction (the affective domain) and Intellectual Accessibility (the cognitive domain). The two aspects of attitude measured by ASCI (V2) are related, though not redundant, which is supported by two-factor confirmatory factor analysis (Xu & Lewis, 2011). The validity of the two-factor correlated structure has been confirmed in subsequent studies (Sen et al., 2016).

#### VSEPR Test Instrument

An 11-item multiple-choice assessment of VSEPR chemistry achievement developed by Merchant et al. (2013) was administered during the pre- and post-test phases of the study. The instrument examines three principles of VSEPR theory: bond angles (items 1−3), molecular geometries (items 4−8), and the identification of shapes of molecules based on their molecular formula (items 9−11). For each of the 11 items, a score of 10 is awarded for a correct response and a score of 0 for an incorrect response. Content validity was confirmed, and Cronbach’s alpha measurements suggest adequate internal consistency (Merchant et al., 2013).

#### Purdue Visualisation of Rotations Test (ROT)

A widely used measure of spatial ability in science education. For this study, the revised 20-question version of the ROT was employed (Bodner & Guay, 1997). Students are allotted 10 min to complete the test. For each question, students are given an example of a rotation on a 3-D object, which then requires the student to perform the same rotation on a different object and choose the correct result from a pool of five options. The test has consistently demonstrated good reliability across several studies (Bodner & Guay, 1997; Rahmawati et al., 2021).

#### ChemFord

A free-augmented reality mobile and tablet application available on Apple iOS (iOS 11.0 or later) and Android (4.4 and up) platforms (Elford et al., 2021).

### Participants and Procedure

**Fig. 1:**
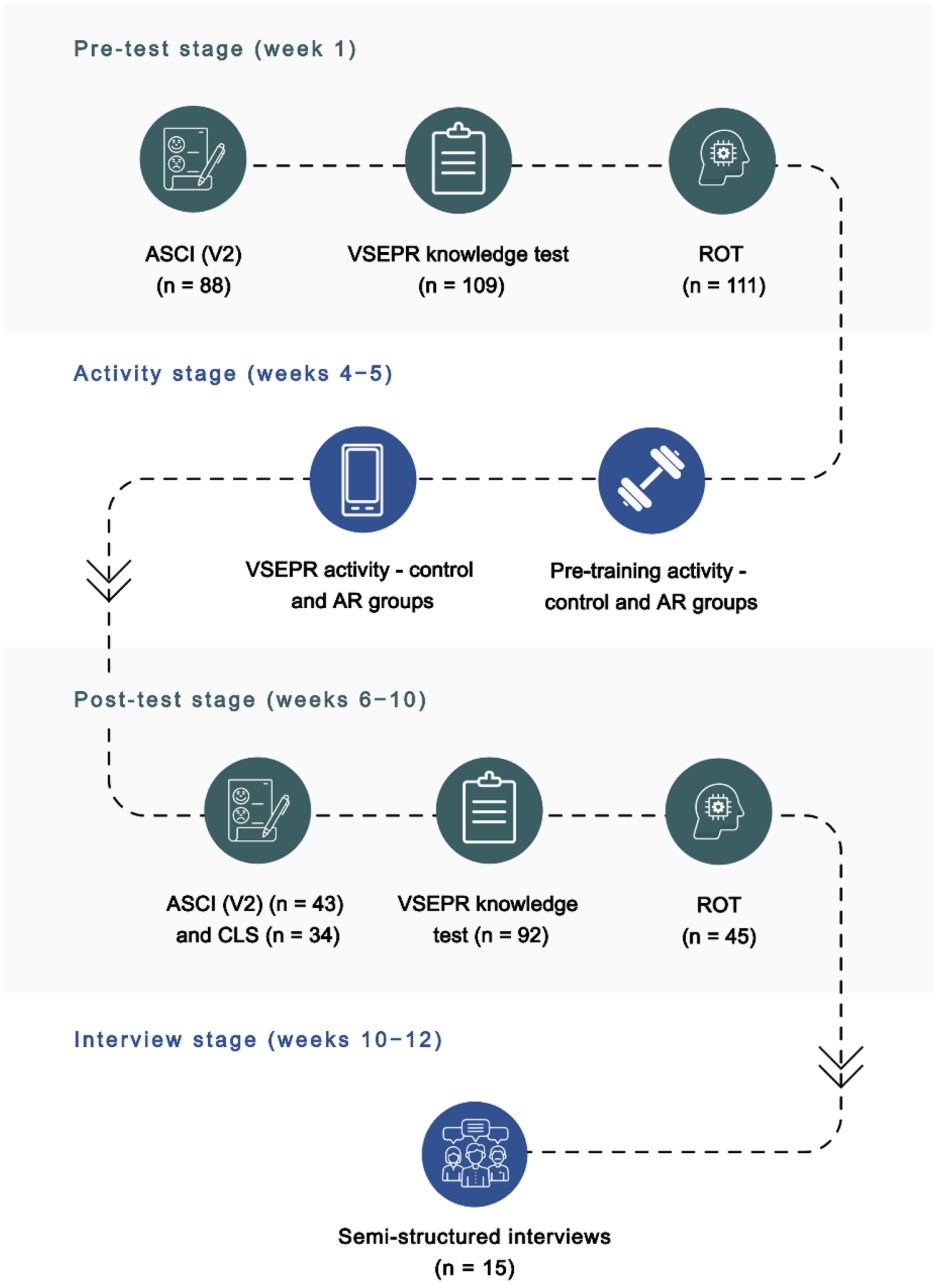
Experimental design utilised for this study, including details of participant engagement

This study was conducted throughout the academic year of 2020/2021 as part of a compulsory module of inorganic and general chemistry study at the University of East Anglia (UEA). The School of Chemistry at UEA is a dual-intensive (research and teaching) department teaching bachelors and integrated masters’ degrees in chemistry. The participant cohort identified for this research were first-year UK higher education students. A pre-test/post-test experimental design (Fig. 1) was employed, and participants were randomly assigned to one of two cohort groups to avoid bias and confounding variables as follows:

1. **Control group:** The learning activity incorporated two-dimensional perspective drawings of different molecular geometries as described by VSEPR theory.
2. **AR group:** The learning activity incorporated image markers from the ChemFord AR application which launched augmented reality models that the students could manipulate (see Supporting Information for application details).

Throughout the study, each group participated in only one learning activity to eliminate carryover effects. A synchronous teaching session was conducted with the student cohort prior to the activity.

### Research Questions

This study attempts to explore how the integration of augmented reality technology into an asynchronous online activity impacts students’ conceptual understanding of VSEPR. Participants’ cognitive load, attitudes, and spatial ability were measured alongside their perceptions of the AR technology and our asynchronous online VSEPR activity. The research questions investigated were as follows:

- ***Research Question 1*.** Does the introduction of augmented reality in an asynchronous online learning initiative improve test performance on the VSEPR test instrument of the AR group compared to the control group?
- ***Research Question 2*.** Do participants in the AR group display different cognitive effects for intrinsic, extraneous, and germane cognitive load compared to the control group?
- ***Research Question 3*.** Does AR result in greater performance gains for students who previously exhibited lower spatial ability?
- ***Research Question 4*.** Do students in the AR group display different responses to the Attitude to the Study of Chemistry Inventory compared to the control group?
- ***Research Question 5*.** What are the students’ perceptions of (i) the implementation of the AR technology and (ii) our asynchronous online VSEPR learning intervention?

### Activity Design

**Fig. 2:**
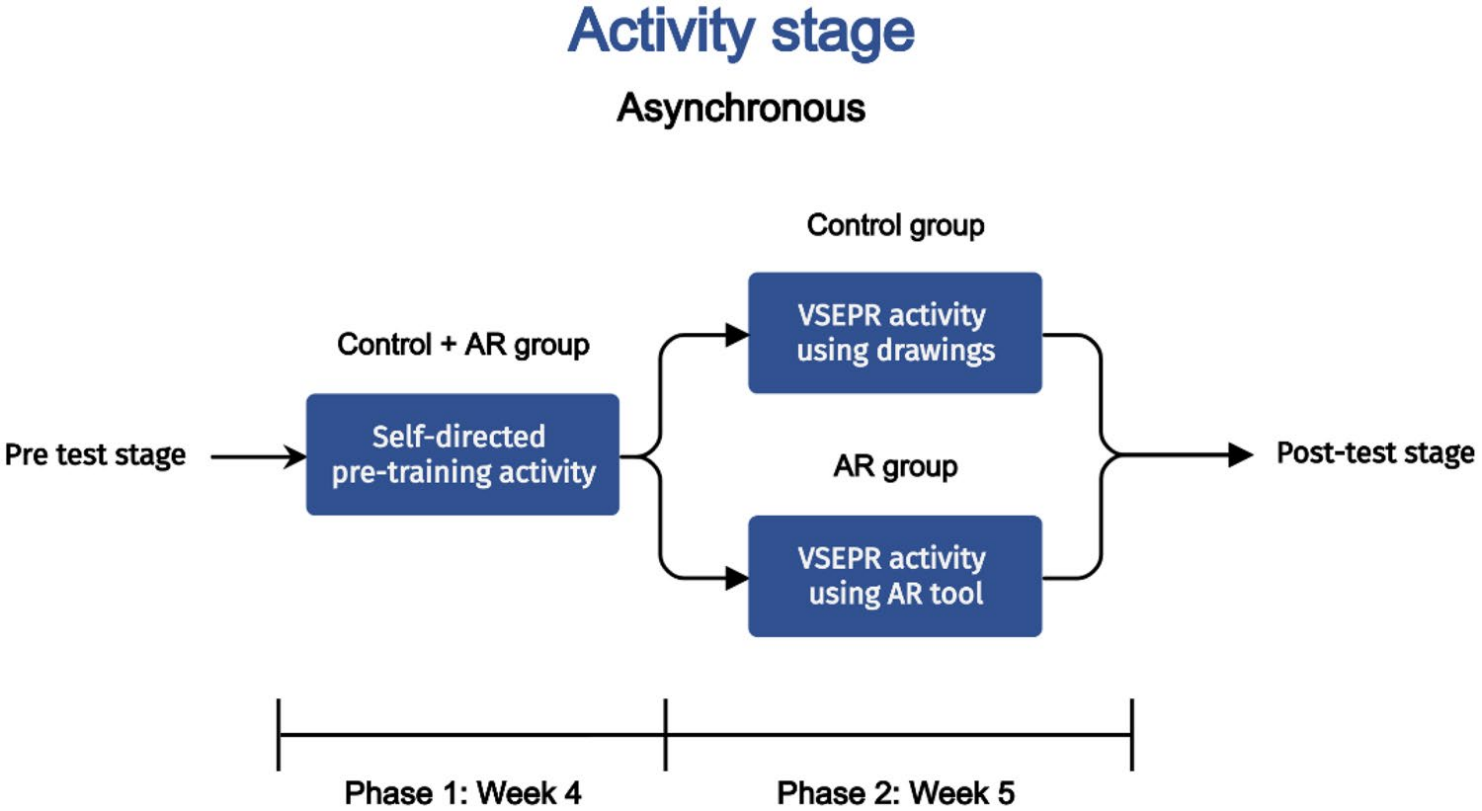
Overview of the activity stage of the study, including details of group allocation

The educational objective of this study was to develop an asynchronous online activity where higher education chemistry students can apply concepts of VSEPR theory to solve engaging problems. The activity stage was composed of two phases (Fig. 2) which were conducted in weeks 4 and 5 of the semester.

Firstly, a pre-training exercise was carried out with students on the topic of the Berry pseudorotation mechanism (Ugi et al., 1971). The pre-training principle states that individuals learn more deeply from multimedia when they know the names and characteristics of a concept (Mayer & Pilegard, 2014; Meyer et al., 2019). This lessens the cognitive load experienced when presented with novel concepts (Mayer & Pilegard, 2014).

To reduce the redundancy (expertise reversal effect) (Kalyuga, 2007) we sought to give autonomy to the learner. To support this, the pre-training was designed as an asynchronous self-directed learning activity. Three principles of the Cognitive Theory of Multimedia Learning guided the pre-training design: (i) the continuity principle, which is to “align words to corresponding graphics” (Clark & Mayer, 2011); (ii) the segmenting principle, which is to break down complex information into smaller sections, which are presented sequentially (Clark & Mayer, 2011); and (iii) the coherence principle, which states that all unnecessary information (extraneous material) should be eliminated (Clark & Mayer, 2011). A copy of the pre-training exercise can be found in the supporting information.

The second phase was an asynchronous online activity embedding elements of game-based learning (Li & Tsai, 2013). We constructed this activity as a gamified approach to engage students with problems based on concepts of VSEPR theory. A copy of this activity can be found in the supporting information. The narrative of the activity places students as part of an expedition to the lost city of “Gillespie”. The ancient inhabitants, “Gillespians”, employed inscriptions based on molecular shapes which students must decipher to safely lead the expedition. To assist them, students are presented with the “Adventurer’s Logbook”.

The logbook provides students with a worked example of how to use the information provided by a Lewis structure to determine the correct corresponding molecular shape. Extensive research has shown that example-problem pair formats are an effective approach to problem solving (Leppink et al., 2014), particularly for the novice. The subsequent pages of the logbook outline four different collections of inscriptions, based on VSEPR theory, that students must correctly evaluate to deduce the correct path. Inscriptions for the control group were supplemented with perspective drawings of molecular geometries, whereas the inscriptions for the AR group used ChemFord image targets for generating the corresponding 3D virtual object. Addressing research question 2, we sought to investigate the impact of the AR visualisation aid on the ECL of the learner.

For this activity, students were required to submit their responses in long-answer format. This proved critical to evaluating whether students demonstrated a deep understanding of the topic material, and were assessed using the measurement rubric in Appendix 2.

## Results and Discussion

This study explored the utilisation of an asynchronous online learning activity which employed elements of gamification and AR technology. Higher education chemistry students apply the concepts of VSEPR theory to solve problems. Descriptive statistics concerning the variables of conceptual understanding, cognitive load, and attitude to chemistry in response to completing our activity are summarised in Table 1. In addition, analysis of students’ spatial ability, plus their perceptions to the AR technology, and our VSEPR intervention, are also provided.

**Table 1:** Relative means and standard deviations for VSEPR knowledge, cognitive load, and attitude to chemistry

Variable	Control group	AR group
	Mean (SD)	Mean (SD)
VSEPR knowledge test score *0 (low) to 110 (high)*		
Pre-test	51.71 (21.55)	47.11 (20.91)
Post-test	71.95 (16.31)	64.47 (18.56)
Cognitive Load Scale responses *(11-point scale)*		
ICL	4.36 (2.11)	5.53 (2.09)
ECL	4.26 (2.37)	4.17 (2.28)
GCL	7.02 (2.46)	6.50 (2.07)
ASCI V2 responses *(7-point semantic differential scale)*		
Emotional Satisfaction	3.92 (1.26)	2.94 (0.72)
Intellectual Accessibility	5.03 (1.09)	4.59 (0.99)

### Analysis of VSEPR Conceptual Knowledge Data

Table 1 shows the relative group-dependent means and standard deviations of the VSEPR instrument test scores obtained before, and after, our activity intervention. Across both groups, 77 students completed the instrument at both the pre- and post-test stages. Following data collection, the Shapiro-Wilk test was used to check for the existence of normality. Although other methods for normality testing exist, Shapiro-Wilk has more power to detect the nonnormality on smaller sample sizes (Gupta et al., 2019). Data was found to be normally distributed for the pre- and post-test stages. In addition, Bartlett’s test was conducted, which verified that the assumption of equal variances was true.

Intergroup comparisons between the two experimental groups showed no significant differences in the pre-test mean scores obtained, *t*(77) = 0.962, *p* = 0.339. In addition, no significant differences were observed in the post-test mean scores achieved by the two groups, *t*(77) = 1.906, *p* = 0.06. However, it is noteworthy that significant intragroup improvements in performance between pre-test and post-test stages were observed for both the control group, *t*(40) = 6.809, *p* < 0.01, and the AR group, *t*(37) = 4.300, *p* < 0.01. This supports the premise that, for both experimental groups, chemistry instruction using a synchronous session coupled with an asynchronous game-based learning activity can enhance chemistry understanding. For this cohort of students, the introduction of AR technologies did not result in a significant improvement in performance on the VSEPR instrument when compared to the control group.

To further investigate the post-test scores achieved by the control and AR groups, an ANCOVA was performed on each of the three sections of the VSEPR test instrument. The experimental group was used as the between-subject factor, with pre-test scores as the covariate. No significant differences were found in student performance on test items pertaining to bond angles, *F*(1,76) = 0.004, *p* = 0.951, and species identification, *F*(1,76) = 0.110, *p* = 0.741. Yet, significant differences were observed for questions regarding molecular geometry, *F*(1,76) = 5.508, *p* = 0.027.

**Fig. 3:**
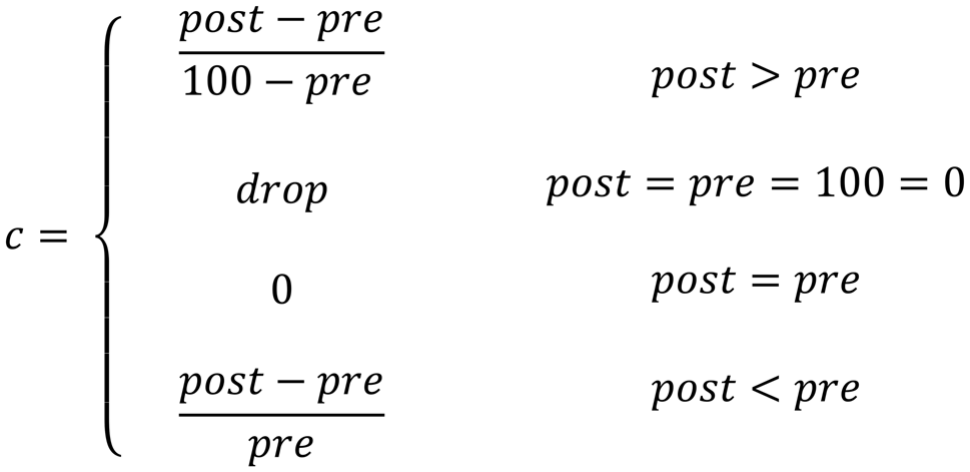
Normalised change calculations (Marx & Cummings, 2007)

Normalised change (*c*) calculations were conducted as a measure of the learning gain of students between the pre- and post-test stages. The higher the normalised change, the greater the learning gain. Marx and Cummings (2007) suggest calculating normalised change as shown in Fig. 3. For this study, the ranges defined by Hake (1998) for normalised gain are adopted: low (*c* < 0.3), medium (0.3 ≤ *c* ≤ 0.7), and high (0.7 ≤ *c*). The *c* values calculated were 0.38 for the control group, and 0.26 for the AR group. To account for the variance in individual scores, the effect size was also calculated. The suggested values for effect size were employed: small (0.2), medium (0.5), and large (0.8) (Wassertheil & Cohen, 1970). The effect size was calculated as 0.36.

To better understand item and scale characteristics of the VSEPR test instrument, the concepts and analytical procedures of Classical Test Theory (CTT) were applied to help determine the overall instrument reliability. CTT is regularly utilised by researchers as the first step in establishing reliability. Its association with basic statistical comparisons means that researchers who have had any exposure to measurement theory are likely to have encountered CTT (DeVellis, 2006). However, CTT has some noticeable shortcomings, such as correlations being computed on the sample which may differ between cohorts. CTT-based methods do not involve the rigorous scrutiny of item characteristics that other methods, such as Item Response Theory, employ.

**Fig. 4:**
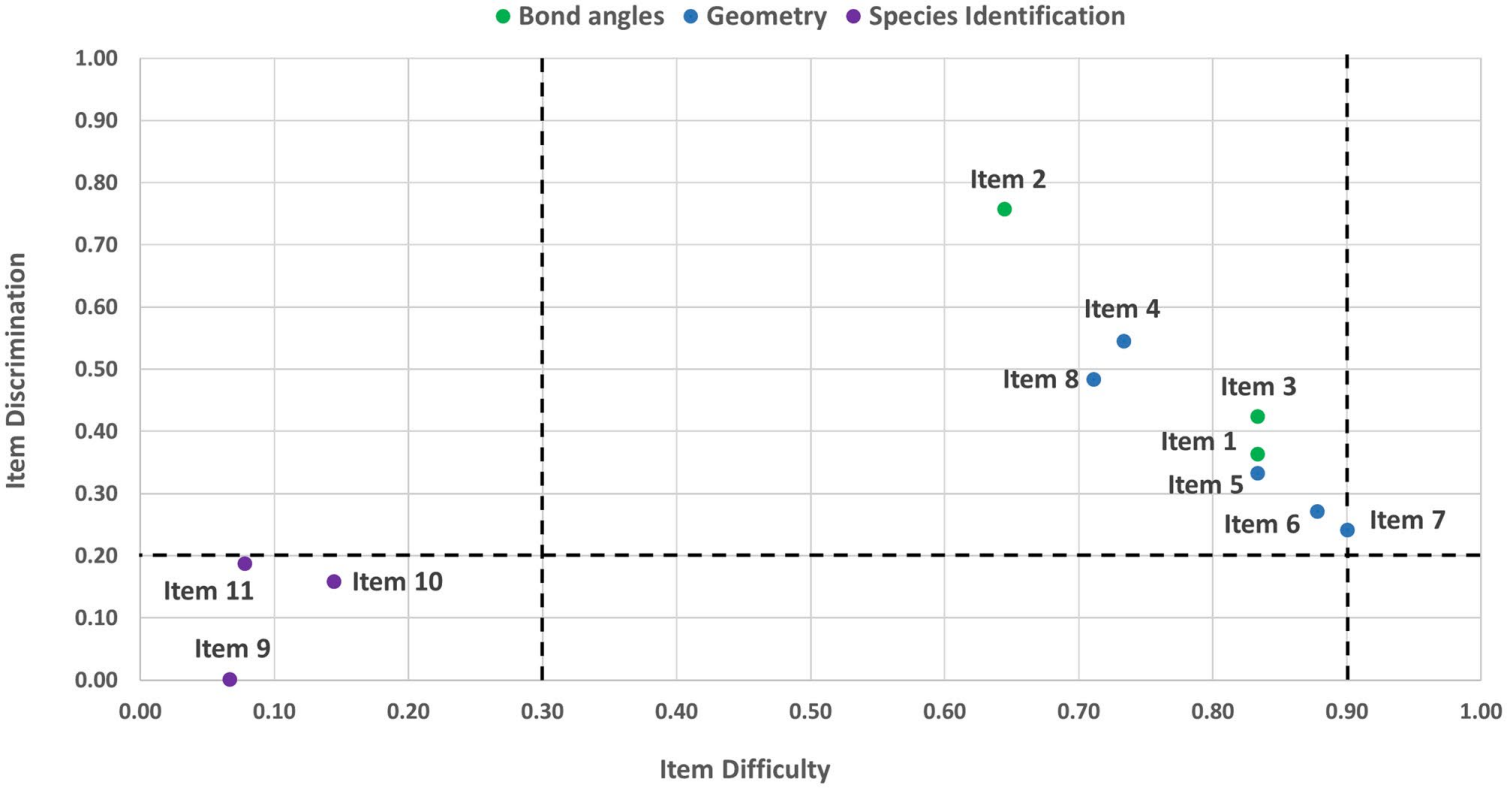
Item difficulty for each of the 11 items in the VSEPR test instrument. The black dashed lines represent the recommended upper and lower bounds of item difficulty and the lower bound of discrimination. Each dot represents an item

Figure 4 shows the calculated properties of difficulty and discrimination for items on the VSEPR test instrument. In the context of educational testing, a difficult item is one that more respondents answer incorrectly. The difficulty values calculated range from 0 to 1, where a higher value indicates an easier item (Kline, 2005). The most effective items have mid-ranges of difficulty (Ding & Beichner, 2009). Yet, in practice, a difficulty of 0.5 on every test item for every cohort is not feasible. As a result, difficulty values within a range of 0.3–0.9 are acceptable (Doran, 1980). Items more strongly correlated with other items, and thus the true score, are fundamentally better items. Such items are said to have greater discrimination (DeVellis, 2006). The extreme group method was used to calculate discrimination with groups partitioned by the top and bottom 27% (Preacher, 2015).

Seven items (2, 4, 8, 3, 1, 5, and 6) fell within the generally accepted range for item difficulty and discrimination. Item 7 falls on the upper limit for item difficulty (implying that it is tending towards being too easy). Items pertaining to species identification demonstrated difficulty values lower than the generally accepted range (indicating harder items) and show low discrimination values. For the remaining items, the discrimination measure ranged from 0.24 to 0.76. Apart from species identification items, discrimination values constitute reasonable evidence that each item’s score is positively related to the overall proficiency represented by total performance on the instrument.

Cronbach’s alpha for total score estimates the internal consistency of the instrument. The alpha value for the VSEPR knowledge test was 0.62, which indicates adequate reliability for an assessment used for low-stake purposes (Cortina, 1993). One item had an alpha-if-deleted value greater than the overall test value: item 9. To see if removing item 9 would improve the reliability, Cronbach’s alpha was recalculated, excluding this item. Cronbach’s alpha for this reduced set of items was 0.66. This suggests that item 9 may not cohere with the rest of the items on the instrument and may need to be reviewed for modification or replacement. However, for the purposes of this study, the original validated instrument (Merchant et al., 2013) was utilised.

**Fig. 5:**
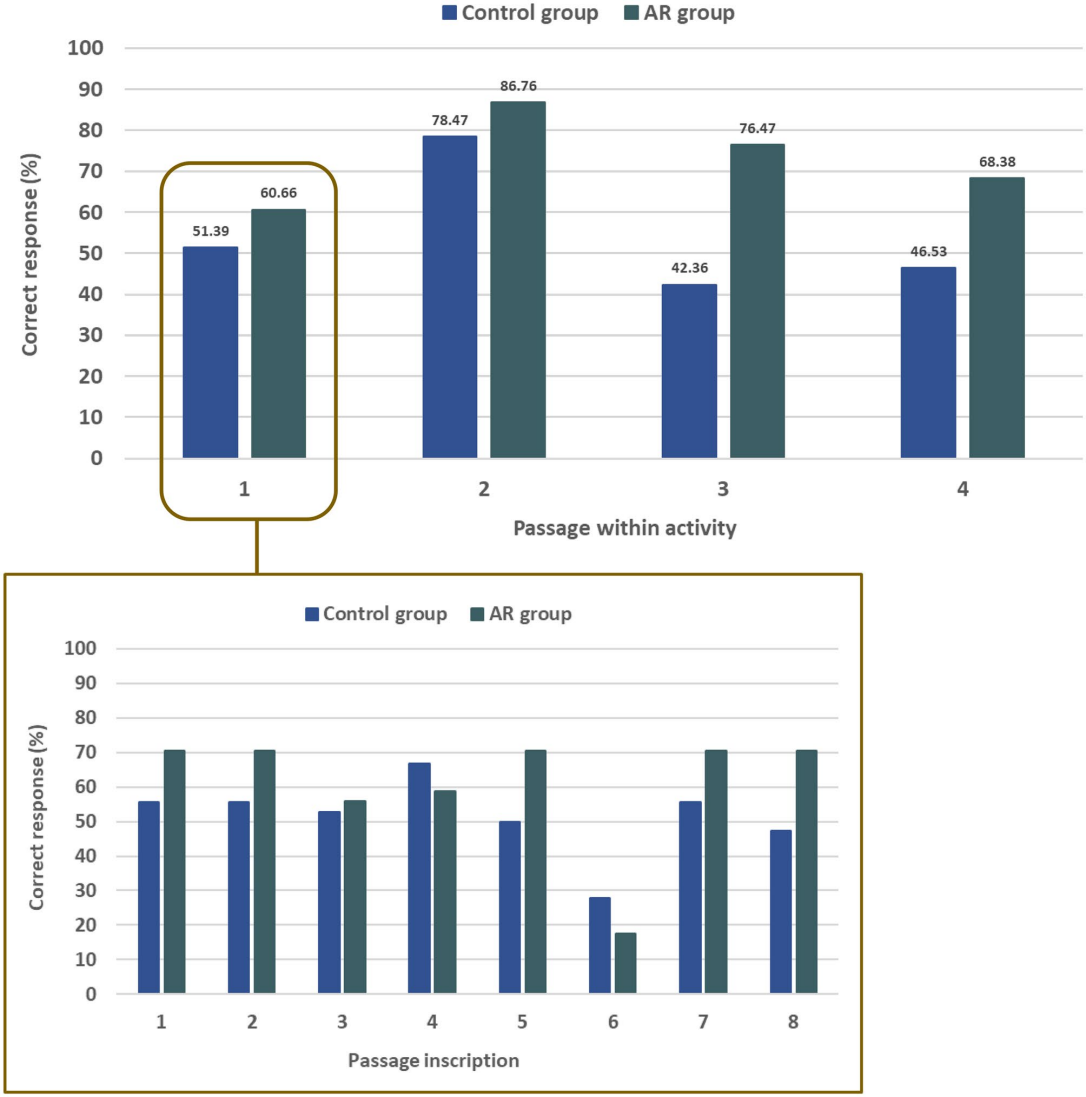
Student scores obtained from our asynchronous online VSEPR activity as guided by the measurement rubric (Appendix 2) for each passage (*top*). The breakdown of passage 1 scores into each constituent inscription (*bottom*)

The analysis of long-answer responses surfaced misconceptions in students’ understanding of VSEPR theory. Following our measurement rubric (Appendix 2), the total score that students could obtain per passage of the online asynchronous activity is equal to *2n*, where *n* is the number of inscriptions per passage. The correct responses (%) achieved by both groups are shown in Fig. 5. Common misconceptions and mistakes identified are summarised as follows:

- A double bond contributes to more than a single bonding group around the central atom (passage 1, inscription 6).
- The “octa” prefix in an octahedral geometry refers to the number of electron groups that can accommodate the central atom (passage 3, inscription 7), rather than the defined vertices of an octahedron.
- Students displayed difficulty describing the shapes of ions (passage 4, inscription 9). Many students could classify ClF_3_ as *T*-shaped, but would incorrectly identify MnCl_5_^2**−**^ as trigonal bipyramidal, thus not accounting for all valence electrons.

Following the pre-training activity, more than 75% of students, from both experimental groups, were able to correctly identify that the Berry pseudorotation occurs in species adopting a trigonal bipyramidal geometry (passage 2, inscription 5). Further, more than 75% of students correctly stated that the Berry pseudorotation does not occur in tetrahedral molecules (passage 2, inscription 6). It was observed that the students in the AR group achieved higher scores on their submitted long-answer responses from the activity than the control group. However, this was not reflected in the post-test performance, where no significant intergroup differences were observed.

### Analysis of Cognitive Load Scale Responses

A total of 34 students completed the CLS instrument. However, responses from 2 participants were incomplete and subsequently excluded from further analysis. To reveal if significant group differences for each type of cognitive load measured were present, an independent sample *t*-test was applied to each of the subscales. No significant differences were detected for ICL, *t*(30) = 1.703, *p* = 0.099, or ECL, *t*(30) = 0.144, *p* = 0.887.

This demonstrates that students perceived that they needed to invest similar levels of cognitive effort to understand VSEPR topic content (ICL), but also to comprehend representations of the molecular shapes (ECL), regardless of whether this was done using the augmented reality tool or perspective drawings. For ICL, this finding is expected, and in line with the meta study by Ibáñez and Delgado-Kloos (2018). The ICL, which describes the complexity of a learning topic itself, should not be influenced by any kind of learning support such as the integration of AR technology.

Our hypothesis was that the introduction of AR would result in a reduction of ECL as students would exert lower levels of cognitive effort to comprehend the representations. Although we did not see this result throughout this study, qualitative data collected from students may offer an insight into why this was the case. Participant interviews suggest that some of the gamification mechanics embedded into the activity required significantly higher effort to overcome relative to the chemistry concepts within the problems. Turan et al. (2016) report that gamification elements occupy the working memory capacities of students, therefore demanding more mental effort. This may have contributed to the ECL of the students, offsetting the cognitive advantages provided by the augmented reality technology.

Furthermore, no significant group differences were observed for GCL, *t*(30) = 0.667, *p* = 0.510. This is reflected in the non-significant difference in mean scores obtained by students on the VSEPR test instrument, in line with the suggestion that GCL is indicative of information retention (Leppink et al., 2013). No significant between-group effect was observed for ANCOVA results when comparing ICL, with pre-test VSEPR test scores as a covariate, *F*(1,29) = 2.721, *p* = 0.103. Lastly, no significant between-group effect was observed for VSEPR post-test scores obtained with GCL as a covariate, *F*(1,29) = 1.799, *p* = 0.190.

### Analysis of Spatial Ability Scores

Conducting tests for assumptions of normality and homogeneity of variances showed a non-normal distribution for spatial ability data collected during both the pre- and post-test stages. As a result, the Levene’s test was used to verify homogeneity of variances (rather than Bartlett’s test) as this is more appropriate for non-normal distributions. A total of 45 students completed both the pre- and post-test spatial assessment. The reliability was measured using Cronbach’s alpha, with values of 0.795 and 0.774 obtained for the pre- and post-test stages, respectively. Intergroup comparisons were conducted using the Mann-Whitney *U* test. For pre-test scores achieved, no significant differences were observed between the two groups (*U* = 250.5, *p* = 0.955). In addition, no significant differences were observed when comparing gender (*U* = 223, *p* = 0.494). However, it should be noted that when comparing gender on all pre-test scores, *n* = 111, a significant difference for gender was observed (*U* = 1108, *p* = 0.043), with males performing better than females. This result is consistent with meta-analysis conducted regarding the correlation of spatial ability and educational performance (Roach et al., 2020).

No significant differences were observed when comparing the post-test scores achieved by the two groups (*U* = 170.5, *p* = 0.06). However, significant intragroup improvements for spatial score were observed for the control group (*Z* = 3.368, *p* < 0.01) and the AR group (*Z* = 3.887, *p* < 0.01) over the course of the study. Spearman’s correlation revealed a ‘moderate’ correlation (*r*_*s*_ = 0.416) between students’ mental rotation ability and VSEPR conceptual knowledge scores obtained, which was found to be statistically significant (*p* < 0.01). A one-way ANCOVA showed no significant differences between-group effects for VSEPR test performance on bond angle determination (*F* = 0.799, *p* = 0.457), recognising geometries (*F* = 2.898, *p* = 0.068), and species identification (*F* = 1.912, *p* = 0.162) using spatial ability as a covariate.

**Fig. 6:**
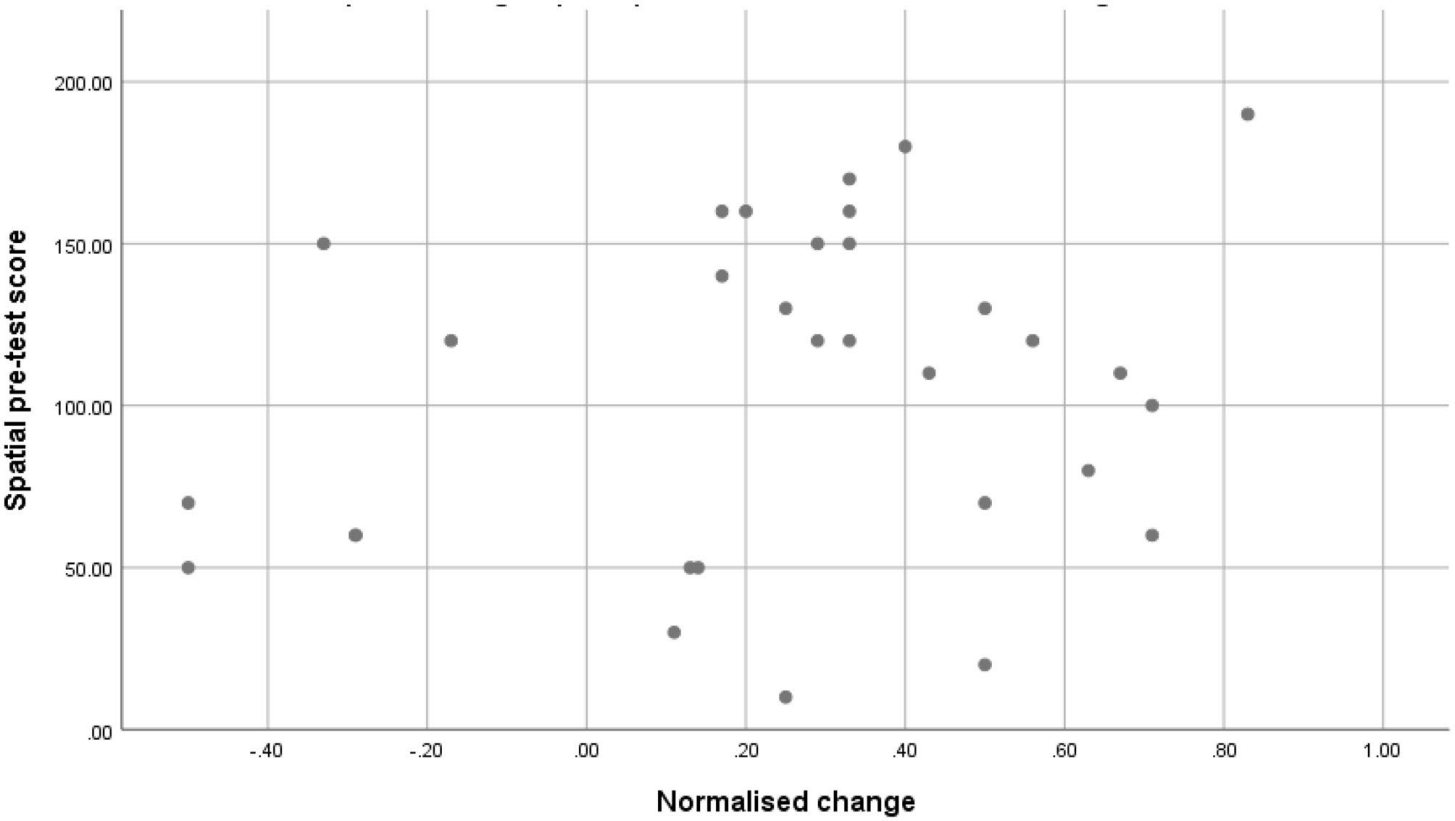
Scatter plot of Spearman’s correlation investigating the relationship between students’ spatial ability and their calculated normalised change in the AR group

To understand if students with lower spatial ability, who utilised AR, demonstrated greater gains in performance, a Spearman’s correlation was conducted between the pre-test spatial scores obtained by students and their calculated normalised change (Fig. 6). This was preferred over other common approaches such as the ‘median split’ to avoid the problems associated with categorising continuous variables (Irwin & McClelland, 2003). No significant relationship was present for this study, (*r*_*s*_ = 0.097, *p* = 0.251), and therefore, further investigation of spatial ability as a predictor of performance gain, through techniques such as regression analysis, was not possible.

### Analysis of Students’ Attitudes

We are interested in exploring relationships between attitude and achievement in chemistry. The questionnaire utilised contains two sub-scales: Emotional Satisfaction (ES), corresponding to the affective domain, and Intellectual Accessibility (IA), representing the cognitive domain. The mean scores reported for each sub-scale by each group are presented in Table 1. In Table 2, we present each item of the ASCI (V2), as reported by both groups, alongside the asymptotic significance, calculated during intergroup comparison (Mann-Whitney *U* test).

**Table 2:** ASCI responses from students in the control group and AR group

Item number*	Sub-scale	Polar adjectives*		Control group: mean (SD)	AR group: mean (SD)	Asymptotic significance
1 R	Intellectual Accessibility (IA)	Hard	Easy	3.73 (1.91)	2.94 (0.93)	.247
2		Complicated	Simple	4.33 (1.40)	2.56 (0.96)	<0.01
3		Confusing	Clear	4.33 (1.44)	4.00 (1.03)	.545
4 R		Uncomfortable	Comfortable	5.00 (1.20)	4.31 (1.20)	.066
5 R	Emotional Satisfaction (ES)	Frustrating	Satisfying	5.27 (1.53)	4.81 (1.47)	.086
6		Challenging	Unchallenging	3.27 (1.75)	2.25 (0.77)	.358
7 R		Unpleasant	Pleasant	5.27 (1.33)	4.81 (1.05)	.281
8		Chaotic	Organised	4.60 (1.96)	4.44 (1.41)	.520

*Items with R were reverse coded during data analysis. Items have been represented in the table in their reverse coded format

The scale reliability was established by calculating the Cronbach’s alpha. For the control group, the alpha values calculated were 0.735 for IA and 0.767 for ES. In addition, alpha values of 0.775 for IA and 0.735 for ES were calculated for the AR group. This indicates, for both groups, that a very good level of internal consistency is present. Interestingly, higher alpha-if-deleted values were calculated with regards to item 8 for both groups. A likely reason for this occurrence is the variance in meanings attributed to these adjectives, which may have resulted in students assigning different meanings to this item. Kahveci (2015) outlines the difficulty in translating item 8 to the Turkish language. It may well be that this item is not consistently interpreted by our students.

Intergroup comparisons show no significant differences for any items from either scale, except for item 2 (Complicated–Simple). When calculating the effect size (Cohen’s *d*) for item 2, a ‘large’ effect size of 1.542 is obtained (*d* > 0.8) (Wassertheil & Cohen, 1970). We hypothesised that AR technology would simplify the visualisation of representations, however, that is not reflected in the ASCI responses collected from both participant groups. Performing a one-way ANCOVA on the VSEPR post-test scores using IA as a covariate shows no statistically significant results. We believe that this difference stems from discussions raised during the qualitative analysis, where students discussed the potential difficulty encountered in overcoming the gamification elements, which confounds with the potential benefits of the AR technology.

### Analysis of Interview Responses

We recruited 15 students, across both experimental groups, to participate in individual semi-structured interviews. The interview schedule covered three topic areas: usability of the ChemFord application (including experience and interaction, and perceived usefulness), the students’ experience of our intervention (including perceived learning effectiveness, satisfaction, performance achievement and reflective thinking), and the cognitive benefits of integrating augmented technologies (including comprehension of topic content, problem solving, and perceived mental effort).

Qualitative analysis of the participant interviews was completed through latent thematic analysis using the approach of Braun and Clarke (2006). Data was recorded, and transcribed verbatim, prior to being subjected to analysis for commonly occurring themes. The initial broad themes were constructed based on frequency and similarity of responses. Redundancy was eliminated and closely related major themes were merged. In this paper we focus on three predominant themes found in student discussions: supporting the learning experience, augmented reality as an asset, and challenges of integration. We report the use of negotiated agreement as the reliability measure for this data set to minimise subjectivity in the coding process and reduce errors. Differences were discussed and where there was a consistent disagreement a common approach was agreed.

### Supporting the Learning Experience

Throughout our discussions, students commented that the examination of molecules within the augmented environment not only reinforced their three-dimensional understanding of the VSEPR concepts, but also helped them appreciate the three-dimensional nature of chemistry, “I always forget that molecules are, you know, three-dimensional... [this] is a constant reminder that we have to think that these are three dimensional molecules.” (Interviewee K).

Students perceived that the integration of augmented reality, as an additional mode of learning, into the teaching process improved their understanding of VSEPR, “With the app, I understand it better than if I was just using paper.” (Interviewee L). Similarly, regarding multiple contexts for learning, “I think ChemFord definitely allows you to see it better... it actually took me quite a long time to grasp what the 2D drawings were actually trying to show.” (Interviewee M). In addition, students commented on their feelings of engagement with the teaching content when AR technology is utilised.

“With the AR, if more of the lecturers did it, I would definitely like it a bit more. It breaks up the teaching content and makes it more interesting.” (Interviewee N).

The ability to manipulate objects within the augmented experience (moving/rotating/scaling) was considered an important affordance of the application, “If you had a molecule that was slightly different so maybe, a mirror molecule to a different molecule, you can always compare by twisting and turning, making it bigger and smaller... And it helps me understand the difference between different molecules in different forms.” (Interviewee D).

Our VSEPR asynchronous online activity was also positively perceived. All interviewed participants expressed a desire to repeat this style of activity in future modules throughout their degree programme. “I would like to see more of these. I’ve just really enjoyed having to challenge myself in a different way.” (Interviewee J). Students frequently stated that the worked example in the ‘Adventurer’s Logbook’ assisted them in correctly identifying the geometry of molecular species within the activity. The majority of students suggested that this recurrence should be once or twice a semester (typically a 12-week period at UEA).

Participants enjoyed the challenge presented by the gamification mechanics embedded into the activity, “It was a really nice change to just questions and bringing that sort of logic and having to think deeper” (Interviewee O). Similarly, “It’s not just the chemistry but also the analytical thinking, thinking about the statements.” (Interviewee B). Students additionally commented that our intervention “made me feel a bit more confident on VSEPR.” (Interviewee D), and that the activity “does help you implement the knowledge that you’ve learned.” (Interviewee G).

Our online VSEPR activity was primarily designed as a group activity. With the transition to online learning in response to Covid-19, we wanted to ensure social interactivity between students, and that this activity was an opportunity for students to collaborate, “So, we did that [the activity] together in person, and having me turn something around orient it [a molecule] to show him what I was thinking. That was when I found it most helpful”. (Interviewee F). Yet, a minority of students commented that they “like doing it [the activity] independently.” (Interviewee L). The design of the activity allows students to utilise skills both working in a team and solving problems independently.

### Augmented Reality as an Asset

A positive opinion ran throughout most participants discussions regarding the AR technology. This positivity was found both in comments regarding the affordances that AR provides, and in remarks about the alternative resources that students purport to use. For visualisation of molecular structures, students commonly mentioned the use of Molymod molecular models (Mollymod.com, 2021). Students stated several benefits of the AR tool over physical models. Two discussion points were *convenience* and *availability*.

“I think the AR can work better. I would have to go out and get the Molymods, whereas I can download the app and have it in 30 seconds. That was preferable.” (Interviewee A).

Convenience was frequently attributed to two predominate discussion points: the ability to generate augmented experiences on their personal mobile devices from a large library of structures and that these structures could be created instantaneously without the additional effort of building the molecular structure. An attributed distinction of the Molymod physical models was the ability to modify the molecular structure to “take molecules apart and build whatever you like. That’s quite useful.” (Interviewee B). This is an affordance not currently provided by the ChemFord AR application.

Students described the user interface of the application as ‘intuitive’, “It’s actually very easy. Very easy to use.” (Interviewee C). This theme was also found throughout a previous thematic analysis of utilising ChemFord for visualising topics of stereochemistry (Elford et al., 2021). As well as these descriptions, further reports of student interaction with the tool suggest minimal frustration experienced by users, an important factor in design of a tool that will be adopted by students.

“I think it’s intuitive. I mean, it always picked up markers quickly. And, you know, just tapping around at the screen, you really quickly figure out how to do stuff on it.” (Interviewee D).

When discussing mental visualisation of structures, in relation to the topic of VSEPR, the role of AR in assisting the visualisation process was of great benefit to students, in comparison to perspective drawings. “If I can see the molecule, that’s a lot better for me. It helps me visualise.” (Interviewee E). Similarly, here: “The app is good for seeing things visually. I don’t really know why I wouldn’t use the app.” (Interviewee F).

### Challenges of Integration

A number of challenges regarding the integration of the ChemFord AR application, and our VSEPR intervention, ran through participants’ discussion. Three major themes evolved from student interviews: (i) exposure of the ChemFord application, (ii) the format of the activity, and (iii) the technological limitations.

Although student comments on ChemFord suggest that it was positively perceived as an educational tool, challenges were expressed regarding integration of the application into the teaching and learning process. Outside of a synchronous learning environment, students explicitly stated reasons why they may not adopt AR technology. Primarily, easy access to the image target library was seen as an obstacle for students.

“If I had the markers to hand, it may have prompted me to look at the shapes. Not having them to hand, I just forgot about it.” (Interviewee G).

Similarly, here,

“I didn't use the AR, just because I didn’t have the markers to hand.” (Interviewee H).

Additional accounts from interviewees describe further reasons attributing to the lower student uptake of this AR technology outside of formal synchronous activities.

“To be honest, once it had been mentioned in lectures you kind of forgot that it was there. So, I just use Google...” (Interviewee I).

In addition,

“I think I would have been more used to using it, but because not all of the lecturers use it. It's kind of like, I haven't been shown it that much.” (Interviewee D).

Participants also expressed the desire to be able to toggle the requirement to scan image targets to generate the augmented experience. As an alternative, students suggested the capability to spawn objects through import from a search function. As such, we have added this as a feature to the application.

“You’re going to need a code, if you want to use it, because if they don’t have anything... I think like maybe add a search bar or something with all the molecules.” (Interviewee E).

“I would like to be able to keep that molecule. So, like, if you scan it could like add it to a database on the app. And you could get back that molecule, get it back up, and without having to scan the QR code.” (Interviewee F).

Recurrent themes of our VSEPR activity were principally coded to (i) difficulty, (ii) gamified elements, and (iii) affective response. Difficulty captured students’ reports of the effort required to correctly apply the VSEPR subject content to evaluate problems. The difficulty of the activity was perceived by the majority of students to be surmountable with a minority commenting that they would have been more satisfied with a harder challenge. “I don’t think it was too bad in terms of difficulty. I thought it was at the right level.” (Interviewee H).

Conversely,

“I just wish it was a little bit harder. It was really interesting and cool, and I’d love to do more things like that.” (Interviewee I).

A minority of students also raised comments regarding device-dependent limitations of their personal mobile device when adopting AR technology, for example, students with devices that do not meet the minimum target API requirements for AR. An important step for integration of this paradigm will be to ensure accessibility for all students whilst keeping up with the rapid pace of technological developments.

### Study Limitations

Some limitations of this study must be acknowledged. Firstly, a major limitation is the relatively small sample size that the data analysis was based upon. The sample size was the result of modest enrolment compounded by participant dropout between the pre- and post-test stages. Secondly, following the adoption of online learning in response to the Covid-19 pandemic, our VSEPR activity was structured as an asynchronous study activity. Consequently, we did not have the opportunity to observe students’ interactions with the AR technology when participants were completing our asynchronous VSEPR intervention. Lastly, we must acknowledge the possibly of self-selection bias from participants. Students who volunteer for interviews may be different from the rest of the population regarding their communication ability or reasoning level.

## Conclusions

This study contributes to the growing body of evidence on how students engage with embedded AR technologies. In summary, a positive opinion of our activity, and the embedded AR technology, ran throughout most participants’ discussions. Students stated that the integration of AR, as an additional mode of teaching, improved their understanding of VSEPR subject content. During the activity stage of this study, participants from the AR group scored higher on submitted answers using our measurement rubric. However, this was not reflected in the post-test. Intergroup comparisons showed no significant differences on VSEPR test instrument performance. In fact, the control group was statistically better on items pertaining to molecular geometry. Further, students from both groups scored low on species identification items. Initial CTT analysis identified items pertaining to species identification as poorly discriminating and hard in terms of difficulty.

Following the activity, responses on the attitude instrument employed during this study showed that the groups scored significantly differently on item 2 of Intellectual Accessibility (Complicated–Simple). The effect size was greater than 1 standard deviation. No further significant differences in students’ responses on the attitude instrument were observed.

When discussing mental visualisation of structures, in relation to the topic of VSEPR, the role of AR in assisting the visualisation process was perceived to be of great benefit to students. However, no significant differences were detected between groups for ICL, ECL, and GCL. We suspect that difficulty stemming from the gamification elements confounded with the potential benefits of the AR technology. The difficulty of the activity was perceived by the majority of students to be appropriate with a minority commenting that they would have been more satisfied with a harder challenge.

Both groups demonstrated significant improvements in spatial ability over the study period, with no significant differences observed in terms of gender performance for the post-test scores. Again, intergroup comparisons did not show any significant differences between groups. A moderate correlation was found between spatial ability and VSEPR test instrument performance.

## Supporting Information

The image marker library for ChemFord can be found at: https://elforddaniel93.wixsite.com/chemford/markers

The ChemFord augmented reality app was not the first to display molecules. However, it has been built by chemists who understand the value of being able to render multiple molecules to explore superposition or intermolecular packing. We are responsive to suggestions from the user base for additional content.

### Electronic supplementary material

Below is the link to the electronic supplementary material.

Supplementary file1 (PNG 694 KB)

Supplementary file2 (PDF 1846 KB)

Supplementary file3 (PDF 2084 KB)

Supplementary file4 (DOCX 17 KB)

Supplementary file5 (PDF 944 KB)

## Appendix 1 Adaptation of the CLS

**Table:**

**#**	**Original item (Leppink**, 2013)	**#**	**Adapted item**
1	The topic/topics covered in the activity was/were very complex	1	The topic/topics covered in the activity was/were very complex
2	The activity covered formulas that I perceived as very complex.	2	The activity covered molecular representations that I perceived as very complex
3	The activity covered concepts and definitions that I perceived as very complex.	3	The activity covered VSEPR concepts and definitions that I perceived as very complex
4	The instructions and/or explanations during the activity were very unclear.	4	The instructions and/or explanations during the activity were very unclear
5	The instructions and/or explanations were, in terms of learning, very ineffective.	5	The instructions and/or explanations were, in terms of learning, very ineffective
6	The instructions and/or explanations were full of unclear language.	6	The instructions and/or explanations were full of unclear language
7	The activity really enhanced my understanding of the topic(s) covered.	7	The activity really enhanced my understanding of the topic(s) covered
8	The activity really enhanced my knowledge and understanding of statistics.	8	The activity really enhanced my knowledge and understanding of molecular geometry
9	The activity really enhanced my understanding of the formulas covered.	9	The activity really enhanced my understanding of the molecular representations covered
10	The activity really enhanced my understanding of concepts and definitions.	10	The activity really enhanced my understanding of VSEPR concepts and definitions

## Appendix 2 Activity measurement rubric

**Table:**

Score given (per inscription)		Explanation (student writes about)		
2	Same *(correctly marked as true/marked as false with correct explanation)*	Passage 1	Inscription 1	H_2_O and SCl_2_ are both bent/angular and exhibit dipole moments
			Inscription 2	Geometry is known as bent/angular
			Inscription 3	BeCl_2_ is linear
			Inscription 4	Lone pairs repel more strongly than bonding pairs
			Inscription 5	Geometry can have a steric number of 3 or 4
			Inscription 6	Double bonds contribute one bonding group
			Inscription 7	Bond angles in bent/angular are less than those in linear geometries
			Inscription 8	Adding a bonding group would result in a trigonal pyramidal geometry
1	More or less *(correctly marked as false with no explanation)*		Student response does not include what is stated above/insufficient explanation	
0	No/wrong answer		Blank/no evidence of reasoning about the question	
2	Same *(correctly marked as true/marked as false with correct explanation)*	Passage 2	Inscription 1	Equatorial groups are separated by an angle of 120°
			Inscription 2	Equatorial and axial groups are separated by a bond angle of 90°
			Inscription 3	The equatorial and axial groups are not equivalent
			Inscription 4	Trigonal bipyramidal has 5 bonding groups
			Inscription 5	This geometry exhibits Berry pseudorotation
			Inscription 6	Removing a bonding group would result in the seesaw geometry
			Inscription 7	The two axial groups are separated by a bond angle of 180°
			Inscription 8	Geometry is called trigonal bipyramidal
			Inscription 9	PF_5_ and Fe(CO)_5_ adopt this geometry
1	More or less *(correctly marked as false with no explanation)*		Student response does not include what is stated above/insufficient explanation	
0	No/wrong answer		Blank/no evidence of reasoning about the question	
2	Same *(correctly marked as true/marked as false with correct explanation)*	Passage 3	Inscription 1	CH_4_ and CF_4_ both adopt this geometry
			Inscription 2	Bond angles are ~109.5°
			Inscription 3	Symmetrical molecules are non-polar
			Inscription 4	Square planar and seesaw geometries both have 4 bonding groups
			Inscription 5	This geometry has a steric number of four and no lone pairs
			Inscription 6	No Berry pseudorotation is observed in tetrahedral molecules
			Inscription 7	The name of this geometry is tetrahedral
			Inscription 8	CH_3_Cl adopts this geometry and displays a dipole moment
			Inscription 9	Phosphate ion is tetrahedral
			Inscription 10	Bonding groups are each located at the corner of a tetrahedron
1	More or less *(correctly marked as false with no explanation)*		Student response does not include what is stated above/insufficient explanation	
0	No/wrong answer		Blank/no evidence of reasoning about the question	
2	Same *(correctly marked as true/marked as false with correct explanation)*	Passage 4	Inscription 1	This geometry is known as octahedral
			Inscription 2	Equatorial groups are separated by a bond angle of 90°
			Inscription 3	SF_6_ adopts an octahedral geometry and is symmetrical
			Inscription 4	Octahedral molecules do not exhibit Berry pseudorotation
			Inscription 5	Axial groups are separated by 180°
			Inscription 6	Octahedral molecules have a steric number of 6
			Inscription 7	Octahedral molecules can only have up to 6 electron groups
			Inscription 8	Replacing axial groups with lone pairs gives rise to a square planar geometry
			Inscription 9	ClF_3_ is *T*-shaped and MnCl_5_^2−^ is square bipyramidal
1	More or less *(correctly marked as false with no explanation)*		Student response does not include what is stated above/insufficient explanation	
0	No/wrong answer		Blank/no evidence of reasoning about the question	
