# Supplementary figures and images for: Exploring the Effect of Augmented Reality on Cognitive Load, Attitude, Spatial Ability, and Stereochemical Perception
Source: J Sci Educ Technol. 2022 Jan 28;31(3):322–39. doi: 10.1007/s10956-022-09957-0 (PMC8795959; doi:10.1007/s10956-022-09957-0)

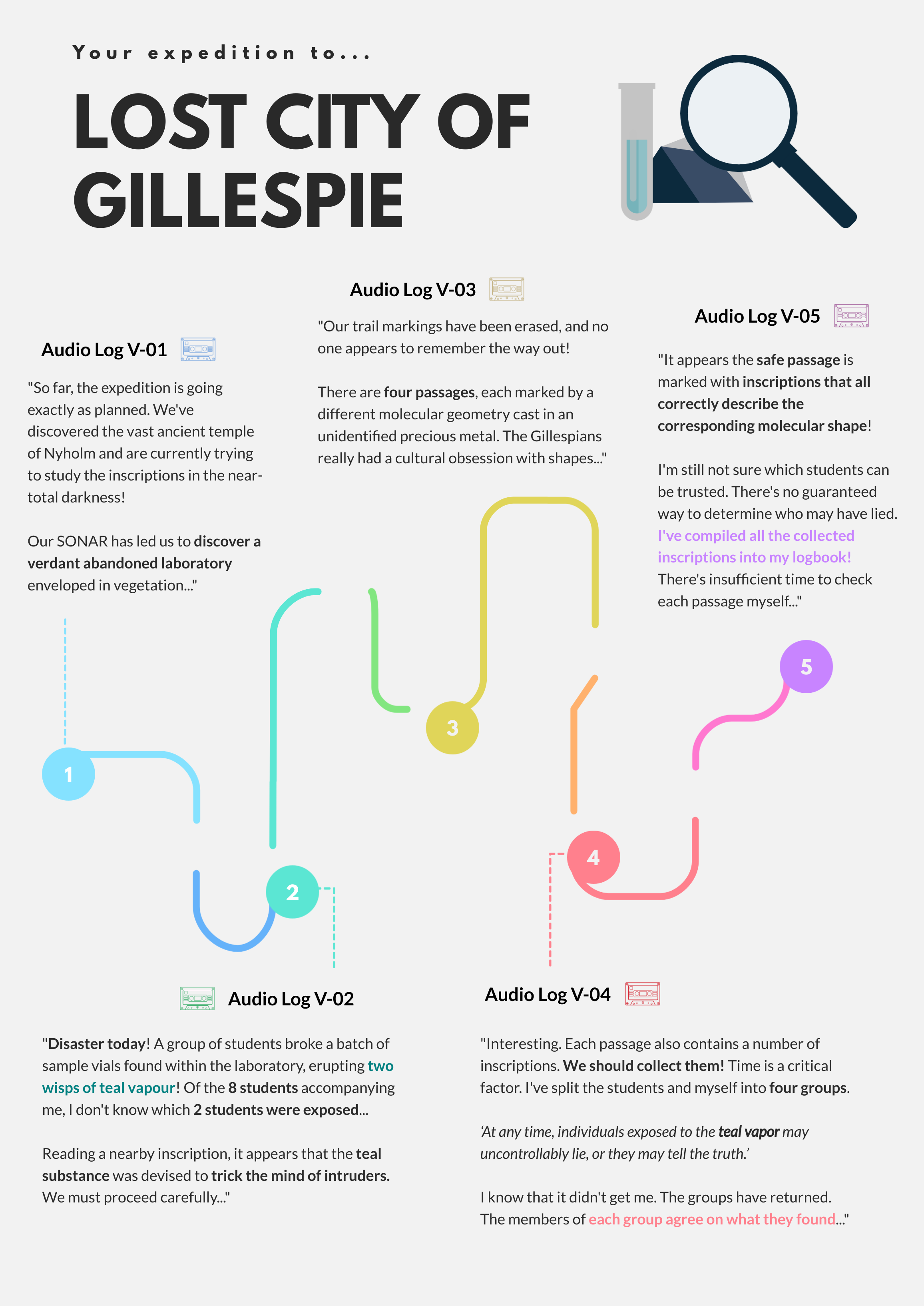

Supplement: Supplementary file 1 — Supplementary file1 (PNG 694 KB) [file 10956_2022_9957_MOESM1_ESM.png]
