# Supplementary material for: Exploring the Effect of Augmented Reality on Cognitive Load, Attitude, Spatial Ability, and Stereochemical Perception
Source: J Sci Educ Technol. 2022 Jan 28;31(3):322–39. doi: 10.1007/s10956-022-09957-0 (PMC8795959; doi:10.1007/s10956-022-09957-0)
Supplement: Supplementary file 2 — Supplementary file2 (PDF 1846 KB) [file 10956_2022_9957_MOESM2_ESM.pdf]

The lost city of Gillespie

LOGBOOK

"...Only one of the passages are safe!"

"Inscriptions can only be true or false...The safe passage is where all corresponding inscriptions are true for the given shape..."

"...Two of the students are under the effect of an unknown teal vapour. They are likely to provide fake inscriptions. I know I can trust my own findings..."

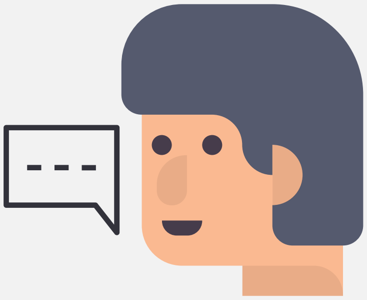

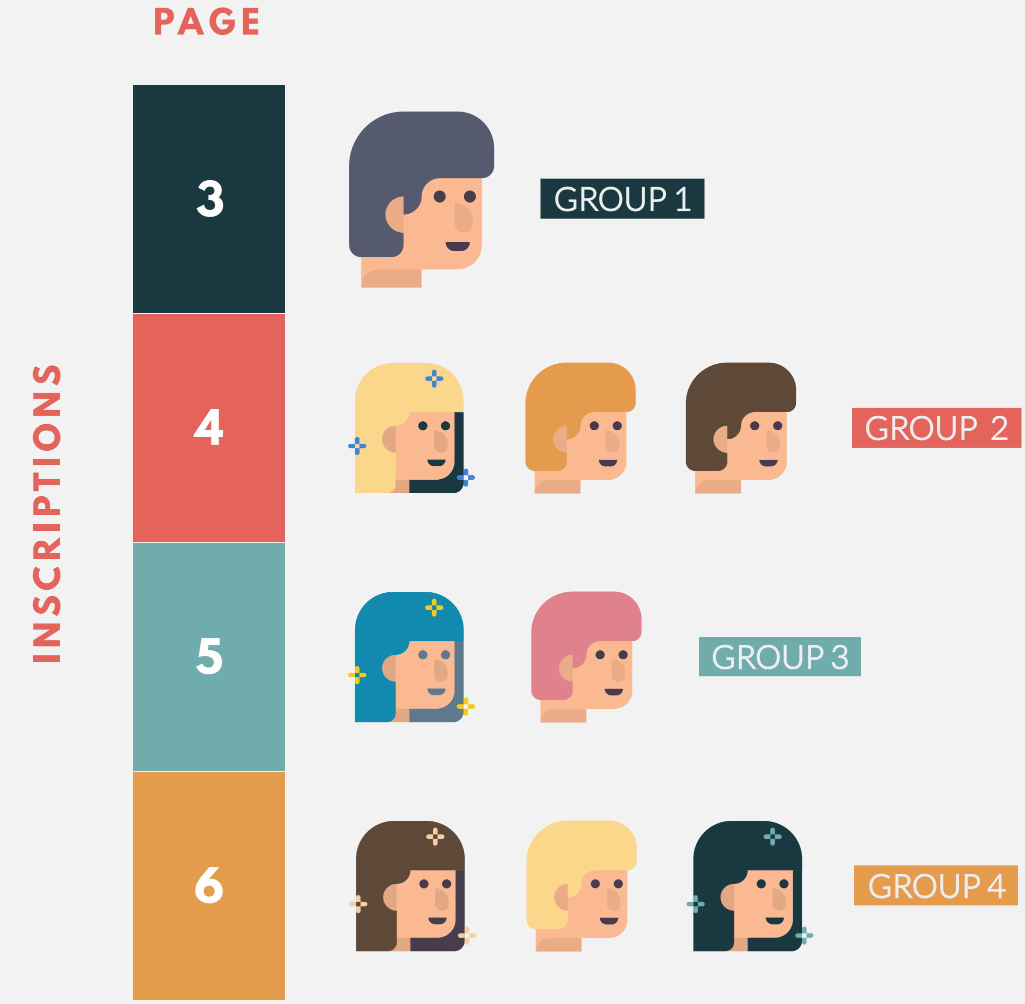

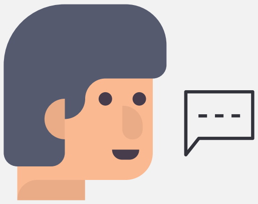

The analysis of the inscriptions is likely to involve the generation of dash-wedge structrures. One approach I could use is shown below!

NH_3

1. Write the chemical formula

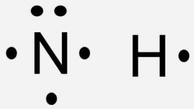

2. Draw the Lewis dot structure of each atom

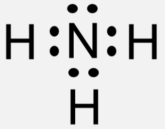

3. Draw the Lewis structure based on the atoms drawn

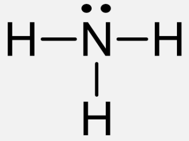

4. Redraw the structure using solid lines

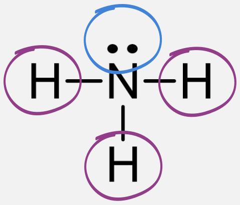

Steric number = 3 + 1

5. Deduce the steric number (Bound atoms + lone pairs)

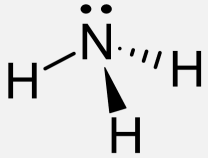

Trigonal pyramidal

AX_3E

6. Draw the correct geometry based on the steric number

Passage 1 Inscriptions

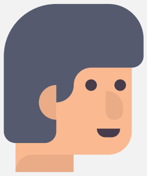

GROUP 1

A = Central atom   X = Bound atoms

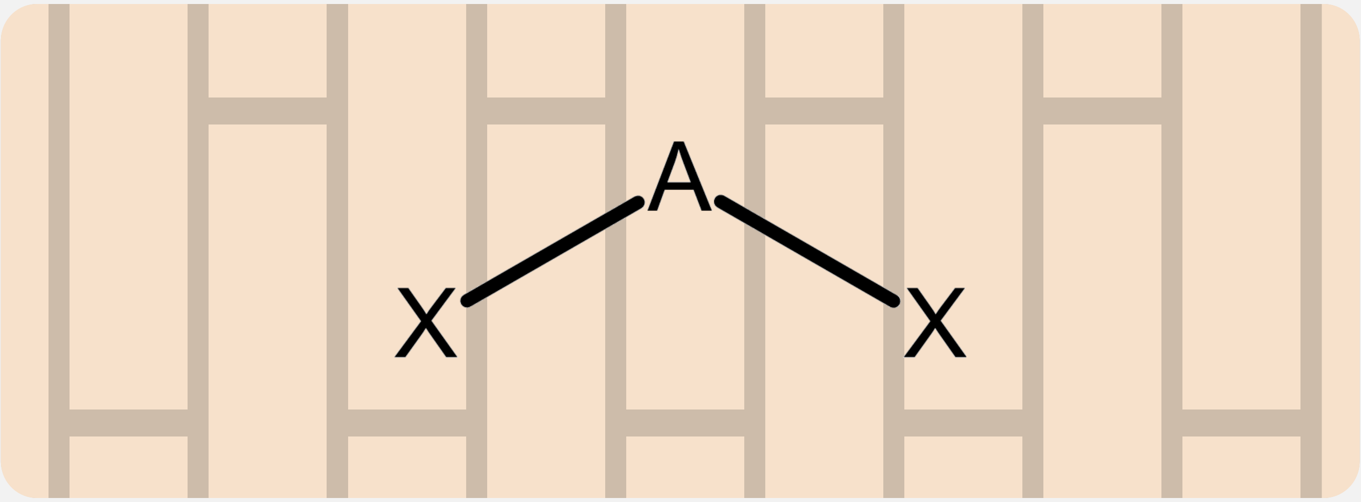

True (T) / False (F)

1. H_2O and SCl_2 both adopt this geometry and exhibit dipole moments... T / F

2. This geometry is known as bent or angular... T / F

3. Substituting S in SCl_2 with Be retains both the geometry and dipole moment... T / F

4. The lone pairs repel less strongly than the bonding groups in this geometry... T / F

5. This geometry can have a steric number of three or four... T / F

6. Ozone and SO_2 do not adopt this molecular geometry as their double bonds contribute more than one bonding group... T / F

7. The bond angles in this geometry are less than those in a linear geometry... T / F

8. Addition of a bonding group would result in a trigonal pyramidal geometry if one lone pair was present... T / F

Passage 2 Inscriptions

GROUP 2

A = Central atom X = Bound atoms

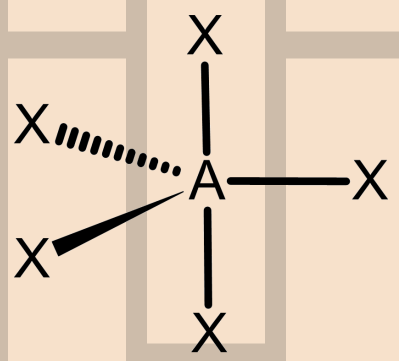

True (T) / False (F)

1. The equatorial bound groups are separated by a bond angle of 120⁰... T / F

2. The equatorial and axial bound groups are separated by a bond angle of 90⁰... T / F

3. The equatorial and axial groups are not equivalent... T / F

4. Within this geometry, there are five bonding electron regions and no lone pairs... T / F

5. This geometry exhibits Berry pseudorotation... T / F

6. Replacing an equatorial bound group with a lone pair in this geometry would result in the seesaw geometry... T / F

7. The two axial bound groups are separated by a bond angle of 180⁰... T / F

8. This geometry is called trigonal bipyramidal... T / F

9. PF_5 adopts this geometry, as does $Fe(CO)_5$... T / F

Passage 3 Inscriptions

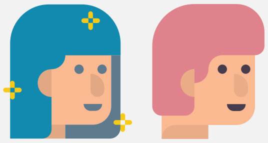

GROUP 3

A = Central atom   X = Bound atoms

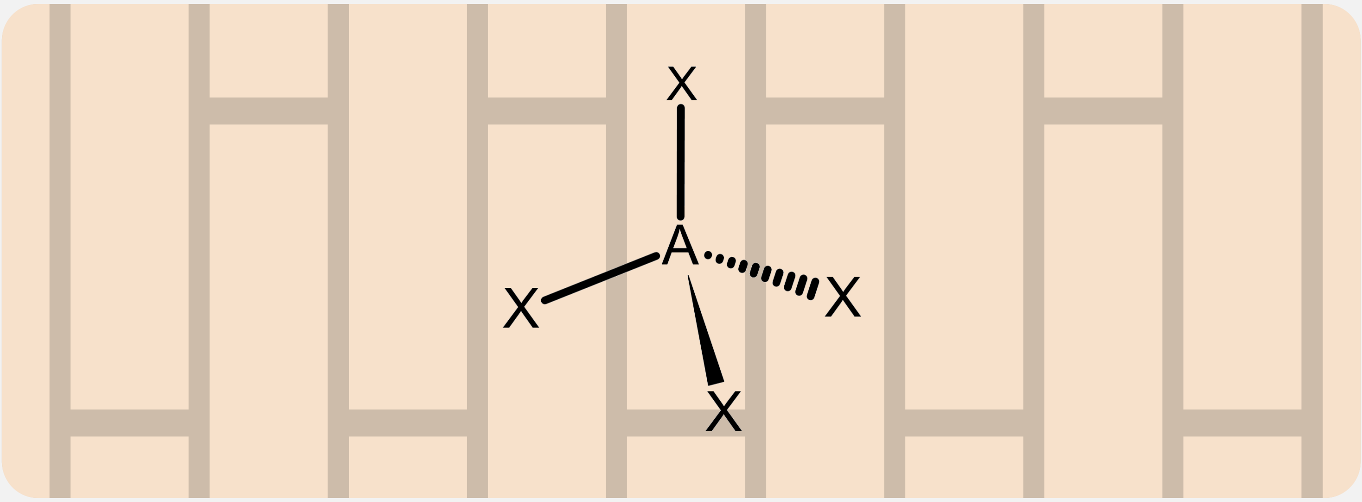

True (T) / False (F)

1. CH_4 and CF_4 both adopt this geometry and are perfectly symmetrical... T / F

2. The bond angle of this geometry is 109.5º... T / F

3. 100% symmetrical molecules of this geometry are always non-polar... T / F

4. Square planar and seesaw geometries have the same number of bonding groups as this geometry... T / F

5. This geometry has a steric number of four and no lone pairs... T / F

6. Berry pseudorotation is not observed in molecules adopting this geometry... T / F

7. This geometry is known as tetrahedral... T / F

8. CH_3Cl adopts this geometry and displays a dipole moment... T / F

9. The phosphate ion adopts this geometry... T / F

10. The shape of this geometry is based on a tetrahedron... T / F

Passage 4 Inscriptions

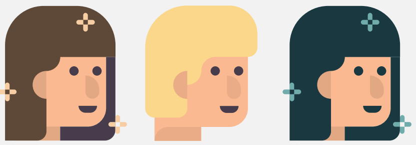

GROUP 4

A = Central atom   X = Bound atoms

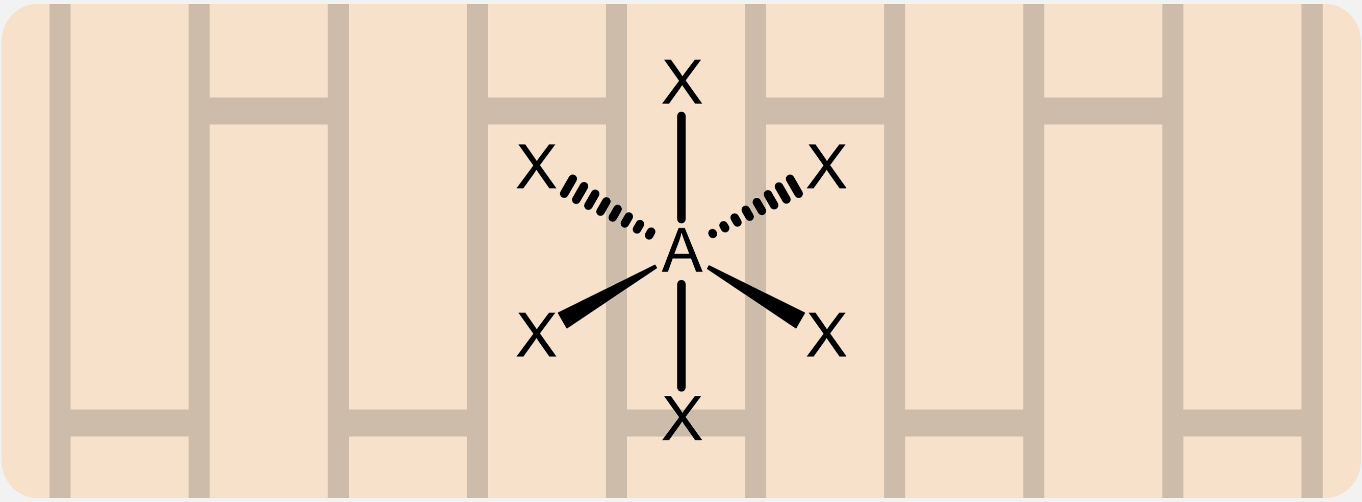

True (T) / False (F)

1. This geometry is known as octahedral   T / F

2. The equatorial groups are separated by a bond angle of 90º...   T / F

3. SF_6 adopts this geometry and is symmetrical, hence exhibiting no net dipole moment...   T / F

4. Molecules adopting this geometry exhibit Berry pseudorotation...   T / F

5. The axial groups are separated by a bond angle of 180º...   T / F

6. This geometry has a steric number of six...   T / F

7. This geometry can have up to 8 electron groups, hence the prefix 'octa'...   T / F

8. Replacing the axial bonding groups of this geometry with lone pairs gives rise to a square planar geometry...   T / F

9. ClF_3 and $MnCl_5^{2-}$ both adopt this geometry...   T / F

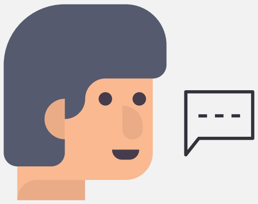

"I should write my analysis of the inscriptions here... This should allow me to work out which passage to take and where the untrustworthy students are...If an inscription is false, I should state why..."

Passage 1 Inscriptions:

Passage 2 Inscriptions:

Passage 3 Inscriptions:

Passage 4 Inscriptions:

Which passage should we take? __________

Extra: Which group has the untrustworthy students? __________

The narrative contains a key clue for this (audio log V-04)!
