# Supplementary material for: Exploring the Effect of Augmented Reality on Cognitive Load, Attitude, Spatial Ability, and Stereochemical Perception
Source: J Sci Educ Technol. 2022 Jan 28;31(3):322–39. doi: 10.1007/s10956-022-09957-0 (PMC8795959; doi:10.1007/s10956-022-09957-0)
Supplement: Supplementary file 4 — Supplementary file4 (DOCX 17 KB) [file 10956_2022_9957_MOESM4_ESM.docx]

Participant Interview(s)

Please see below the schedule used for our semi-structured interviews.

**Usability (Experience and interaction, ease of use, perceived usefulness)**

How easy is the augmented reality intervention to use? What are the best and worst features? What would you improve?

How do you feel this computer program is useful in supporting your learning?

How does the ability to manipulate the objects in real time affect your understanding?

**Cognitive benefits**

How does the augmented reality intervention affect your ability to comprehend the VSEPR topic content? (Was it easier? Better overview?)

How does the augmented reality intervention affect your ability to analyse problems presented on the topic of VSEPR and apply the subject matter?

**THE ACTIVITY:** During the learning activity, how much effort did you need to put into the tasks (the difficulty) and was the way of instruction / content presentation clear?

**Learning experience (Perceived learning effectiveness, satisfaction and performance achievement, reflective thinking)**

Were you satisfied with this type of AR learning experience? Do you believe it would help support your learning achievement?

After using the augmented reality tool, how do you feel you were able to reflect on what you learned?

How did the augmented reality tool help you to identify the main and important issues of the topics?
