# Supplementary material for: Exploring the Effect of Augmented Reality on Cognitive Load, Attitude, Spatial Ability, and Stereochemical Perception
Source: J Sci Educ Technol. 2022 Jan 28;31(3):322–39. doi: 10.1007/s10956-022-09957-0 (PMC8795959; doi:10.1007/s10956-022-09957-0)

Molecular vibrations (trigonal bipyramidal)

Berry mechanism

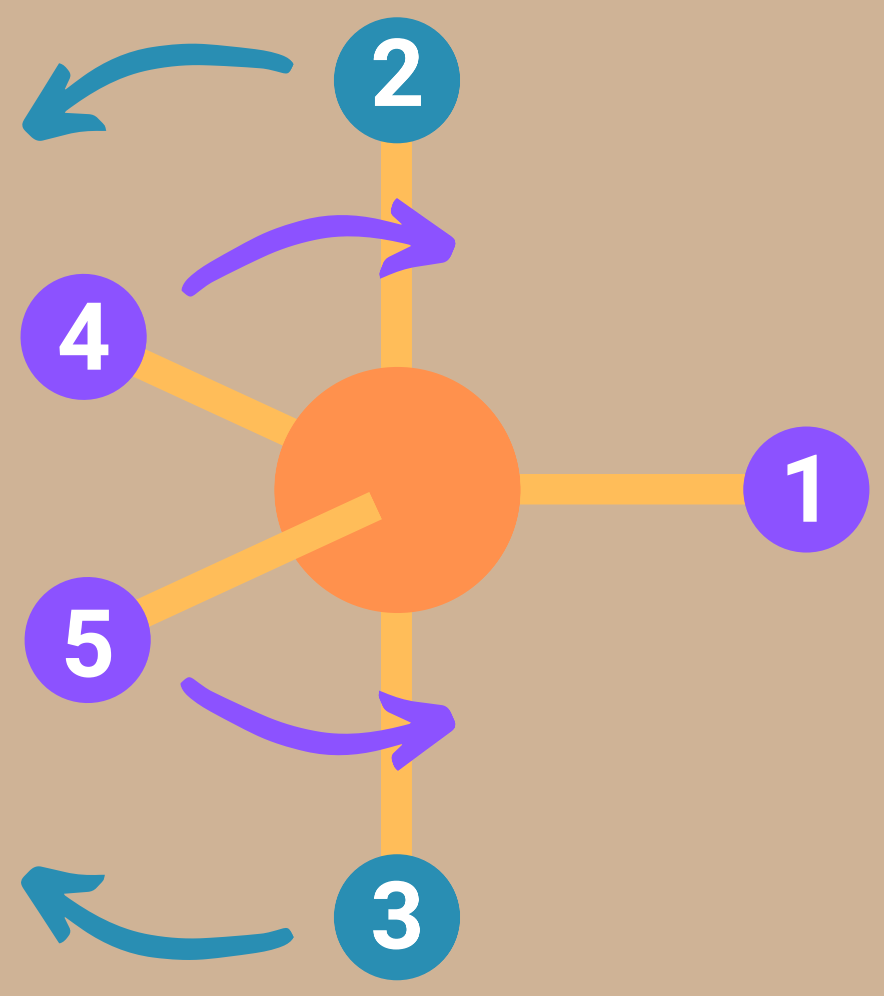

Ligands 2 and 3 move from axial to equatorial positions

Ligands 4 and 5 move from equatorial to axial positions

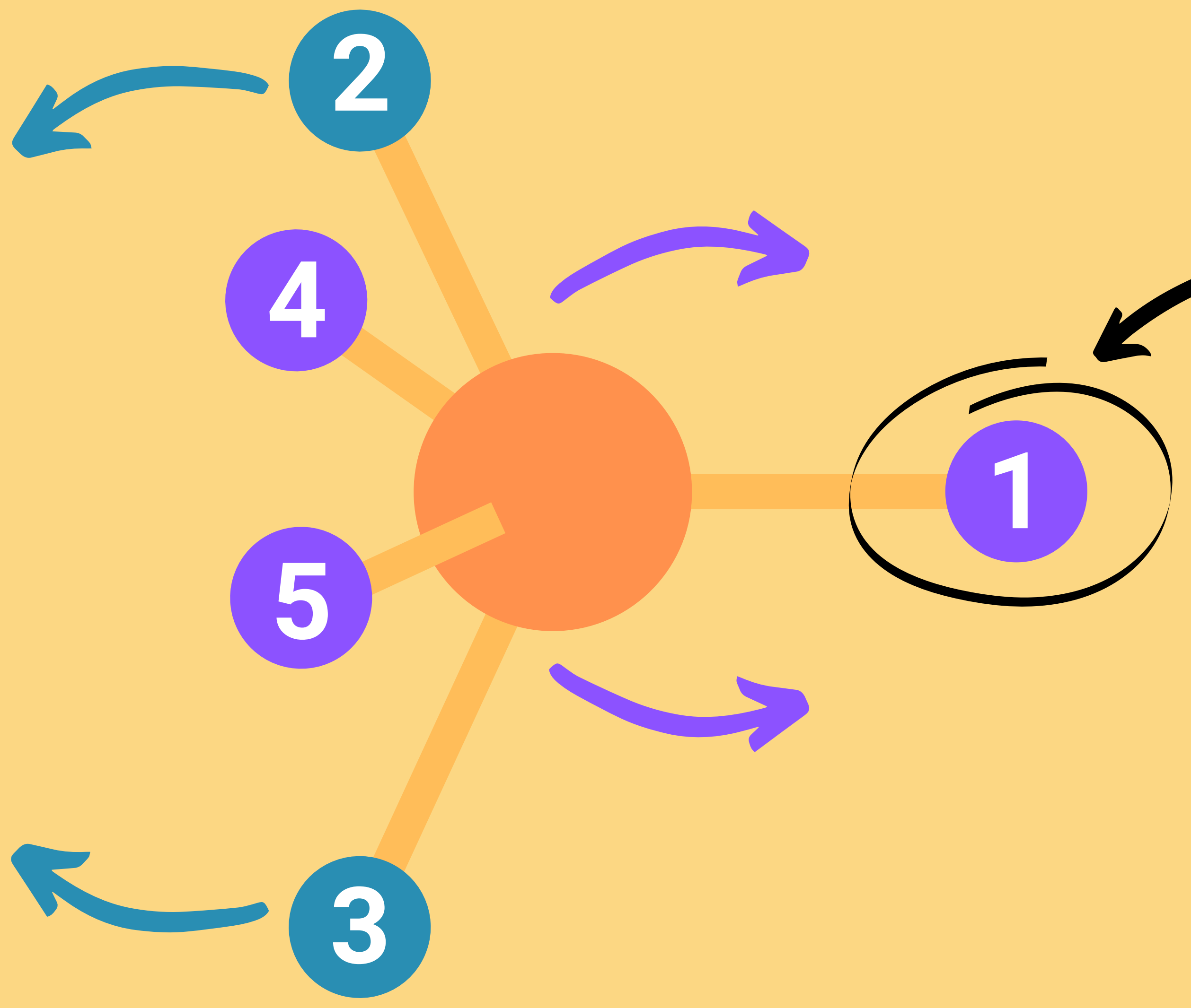

Ligand 1 does not move and acts as a pivot!

At this point, ligands 2, 3, 4, and 5 are all equivalent!
The transition state geometry is therefore square pyramid

The motion is equivalent to a 90 degree rotation around the metal - ligand 1 axis

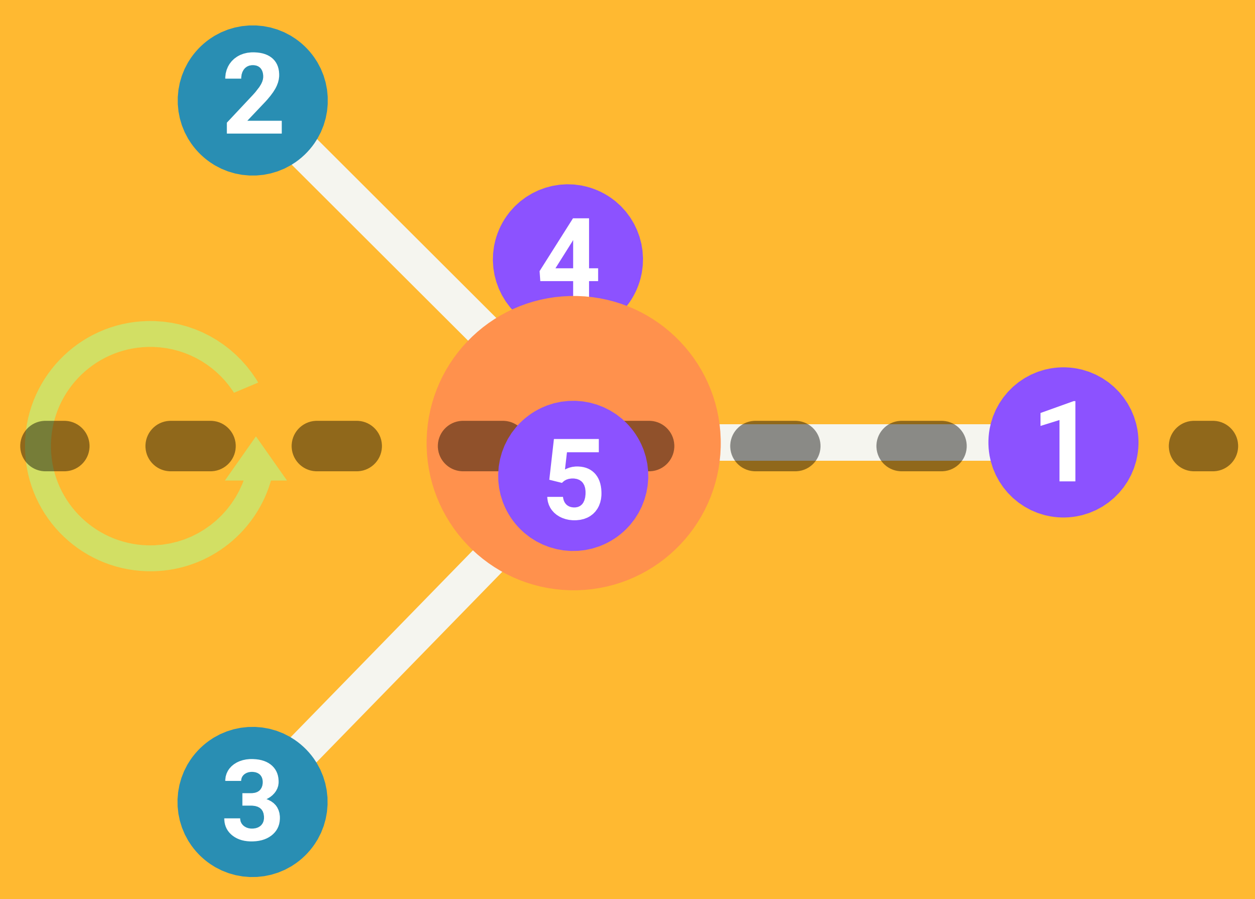

Molecular vibrations (trigonal bipyramidal)

Berry mechanism

Scan me using ChemFord!

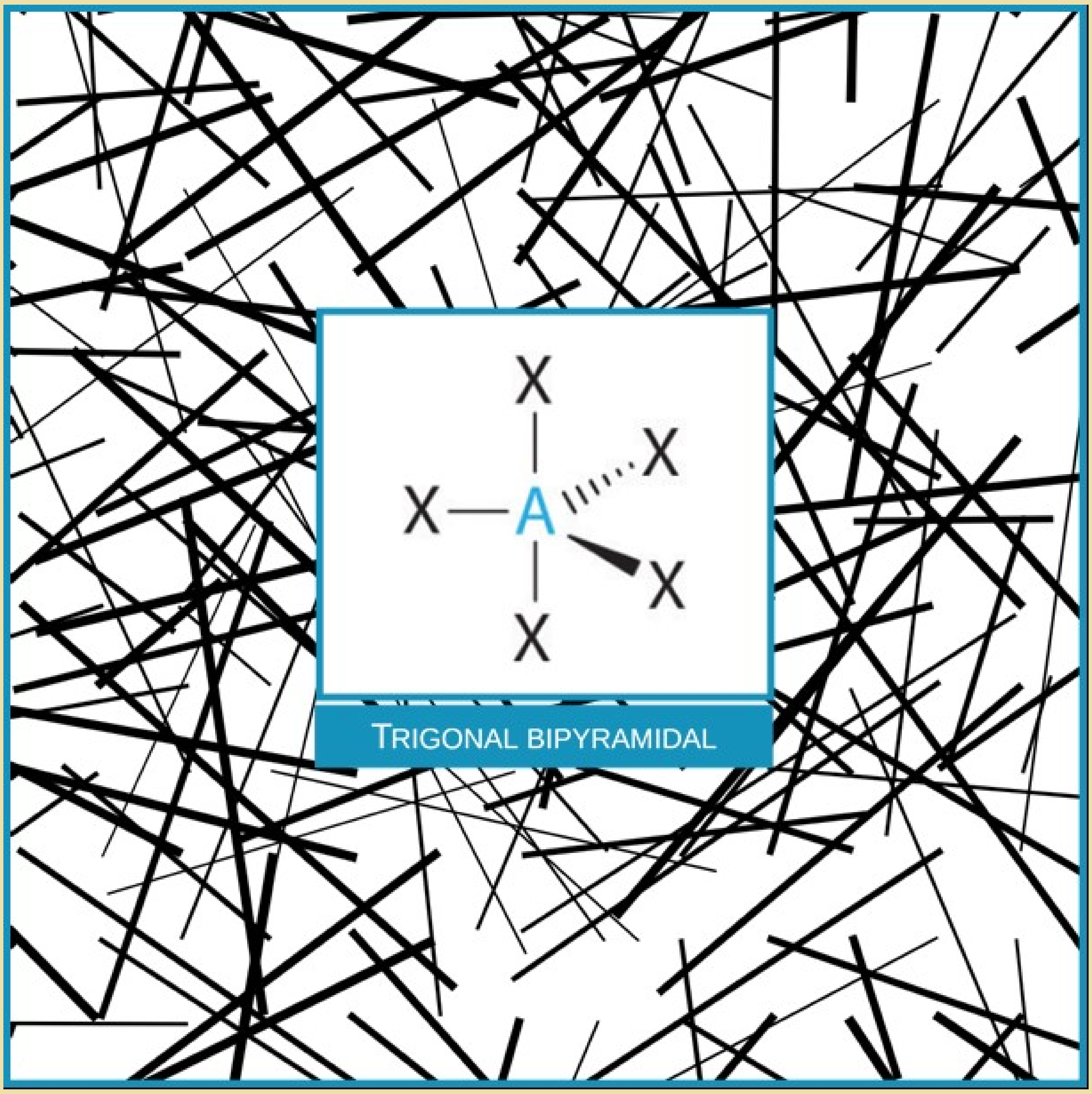

STEP 1
Scan the above image in ChemFord to generate the trigonal bipyramidal geometry (try moving it with your finger!)

STEP 2
Press the "Berry" button to see the mechanism in action!

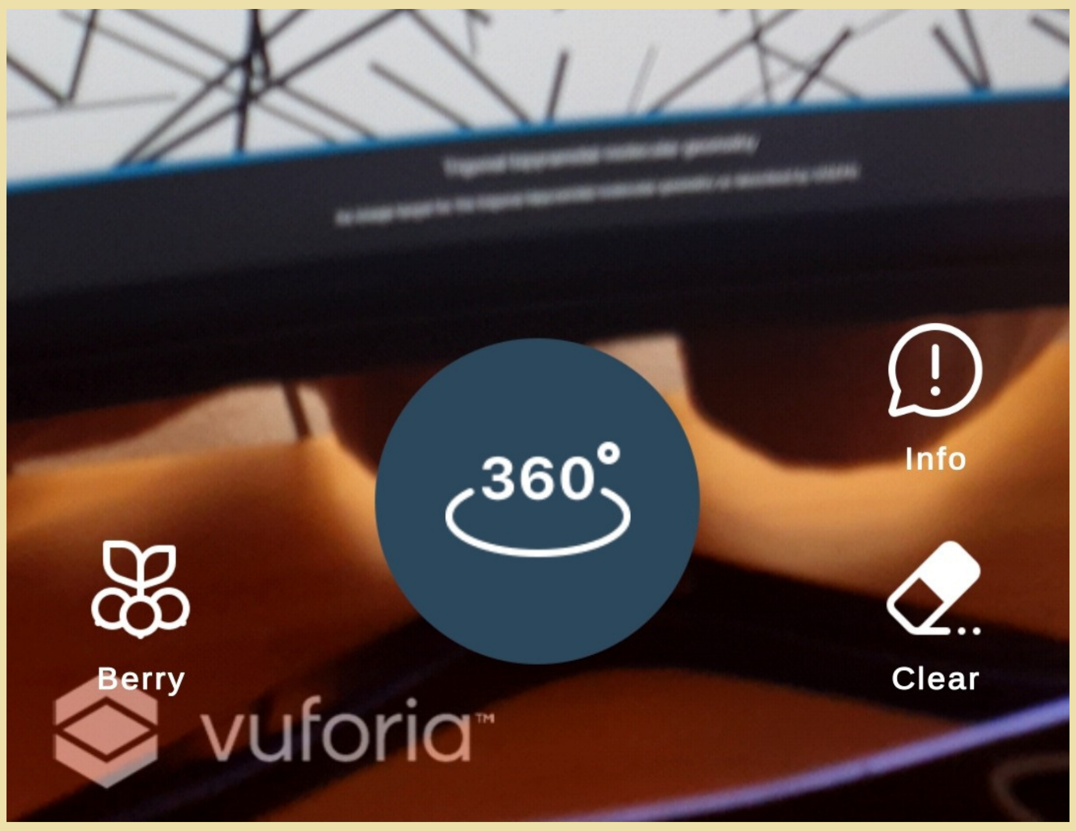

Supplement: Supplementary file 5 — Supplementary file5 (PDF 944 KB) [file 10956_2022_9957_MOESM5_ESM.pdf]
